# Effectiveness of ovarian suspension in preventing post-operative ovarian adhesions in women with pelvic endometriosis: A randomised controlled trial

**DOI:** 10.1186/1472-6874-11-14

**Published:** 2011-05-11

**Authors:** Wee-Liak Hoo, Ertan Saridogan, Alfred Cutner, George Pandis, Davor Jurkovic

**Affiliations:** 1 Department of Obstetrics and Gynaecology, University College Hospital, 235 Euston Road, London, NW1 2BU, UK

## Abstract

**Background:**

Endometriosis is a common benign condition, which is characterized by the growth of endometrial-like tissue in ectopic sites outside the uterus. Laparoscopic excision of the disease is frequently carried out for the treatment of severe endometriosis. Pelvic adhesions often develop following surgery and they can compromise the success of treatment. Ovarian suspension (elevating both ovaries to the anterior abdominal wall using a Prolene suture) is a simple procedure which has been used to facilitate ovarian retraction during surgery for severe pelvic endometriosis. The study aims to assess the effect of temporary ovarian suspension following laparoscopic surgery for severe pelvic endometriosis on the prevalence of post-operative ovarian adhesions.

**Methods:**

A prospective double blind randomised controlled trial for patients with severe pelvic endometriosis requiring extensive laparoscopic dissection with preservation of the uterus and ovaries. Severity of the disease and eligibility for inclusion will be confirmed at surgery. Patients unable to provide written consent, inability to tolerate a transvaginal ultrasound scan, unsuccessful surgeries or suffer complications leading to oophorectomies, bowel injuries or open surgery will be excluded.

Both ovaries are routinely suspended to the anterior abdominal wall during surgery. At the end of the operation, each participant will be randomised to having only one ovary suspended post-operatively. A new transabdominal suture will be reinserted to act as a placebo. Both sutures will be cut 36 to 48 hours after surgery before the woman is discharged home. Three months after surgery, all randomised patients will have a transvaginal ultrasound scan to assess for ovarian mobility. Both the patients and the person performing the scan will be blinded to the randomisation process.

The primary outcome is the prevalence of ovarian adhesions on ultrasound examination. Secondary outcomes are the presence, intensity and site of post-operative pain.

**Discussion:**

This controlled trial will provide evidence as to whether temporary ovarian suspension should be included into the routine surgical treatment of women with severe pelvic endometriosis.

**Trial registration:**

ISRCTN: ISRCTN24242218

## Background

Endometriosis is a common benign condition, which is characterized by the growth of endometrium-like tissue in ectopic sites outside the uterus. The condition is a significant cause of morbidity in women of the reproductive age [1]. Symptoms of endometriosis include dysmenorrhoea, dyspareunia, chronic pelvic pain and subfertility. The revised American Society for Reproductive Medicine (ARSM) classification is the most widely accepted staging system for endometriosis, where a score is used to grade the disease as absent (0), minimal (1-5), mild (6-15), moderate (16-40) or severe (> 40) [2].

Surgical excision of the disease is frequently carried out for the treatment of severe endometriosis. Laparoscopic approach to surgery provides superior views of the pelvis and facilitates complete excision of endometriotic lesions [3]. Although laparoscopic surgery is considered to be less traumatic to pelvic tissues than open surgery, a significant number of women develop post-operative pelvic adhesions even after this form of surgery. The most common site of post-operative adhesions formation is the ovary [4]. Formation of severe post-operative adhesions can compromise the success of surgery for endometriosis by causing chronic pelvic pain, infertility, dysparaeunia and intestinal obstruction [5]. Adhesions formation after laparoscopic endometriosis surgery has been reported in the range of 50 to 100 percent [5-8].

The high incidence of post-operative adhesions in endometriosis patients and their clinical significance underlines the importance of modifying surgical techniques in order to reduce potential adhesion formation. Intra-peritoneal administration of anti-adhesive solutions (e.g. icodextrin, hyaluronic acid) and drugs (e.g. steroids, heparin) at the time of surgery has been advocated as a way of reducing the incidence of post operative adhesions. Currently, only anti-adhesive fluids containing hyaluronic acid have showed evidence of reduction of adhesions however, more studies will be needed to confirm this [9]. Adjuvants have also been suggested to further improve the adhesion reduction, however to date, the most effective product for prevention of postoperative adhesion is yet to be discovered [10].

### Ovarian suspension

Ovarian suspension is a simple procedure which has been used to facilitate ovarian retraction during surgery for severe pelvic endometriosis without any reported complications [11]. A study by Abuzeid et al. showed that temporary ovarian suspension following surgery for severe endometriosis may lead to a reduction in post-operative adhesions [12]. A small retrospective study also found significant reductions in the number of women with post-operative adhesions following ovarian suspension in comparison with data from the literature [13].

### Objective

The aim of this study is to assess the effect of temporary ovarian suspension following laparoscopic surgery for severe pelvic endometriosis on the prevalence of post-operative ovarian adhesions.

## Methods

This is a prospective double blind randomised controlled trial which will be conducted at the University College London Hospital Endometriosis Centre. This centre is a tertiary referral unit for women with severe pelvic endometriosis and receives patients from a wide area of south east England.

### Inclusion criteria

Women age 19 years or older who are diagnosed with severe pelvic endometriosis on pre-operative transvaginal ultrasound scan would be invited to join the study. Suitability for randomisation will be determined at surgery. Only women with severe endometriosis requiring extensive dissection of both pelvic side walls and/or rectovaginal space with preservation of the ovaries and uterus will be included in the study.

Exclusion criteria are inability or unwillingness to provide written consent, inability to tolerate a transvaginal ultrasound scan, unsuccessful surgeries and in cases of complications such as unplanned oophorectomies, bowel injuries or conversions to open surgery.

### Interventions

During laparoscopic treatment for severe endometriosis, both ovaries are routinely suspended to the anterior abdominal wall using a Prolene suture, which is brought out onto the skin and secured using a fine haemostat or 'mosquito' clip during surgery. This is performed to facilitate access to the pelvic side walls during surgery and a complete excision of the disease.

At the end of the operation, women will be randomised to have one ovary suspended for 36 to 48 hours post-operatively. One of the two ovarian suspension sutures will be cut to allow that ovary to fall back into the lesser pelvis. A new transabdominal suture will be reinserted at the same site to act as a placebo. The pneumoperitoneum will then be deflated and the Prolene stitch of the suspended ovary will be tightened with a surgical knot placed over the skin to secure the ovary to the abdominal wall. This will ensure that the suspended ovary is lifted as far away from the pelvis as possible. The surgical knots will be secured with the space of a Carless scissor between the skin and the knot to allow easier removal of the suture and reduce patient discomfort. All randomized patients will therefore have two abdominal sutures of similar length and both the patient and clinical staff - apart from the surgical team - will be blinded to the randomisation process.

A label will be attached to the operation notes to define a) the randomisation number b) the operation date and time and c) the time to remove the sutures. There will be no documentation of the randomisation site in the operation notes.

Both sutures will be cut 36 to 48 hours after surgery by a ward nurse who will not be part of the study and will be blinded to the ovarian suspension site. The only members of staff who will be aware of the site of ovarian suspension will be the surgeons who will be under strict instructions not to discuss individual patient's treatment allocations with the patient or any other members of the clinical and nursing staff.

### Randomisation

Participants will be randomised to unilateral suspension of either the right or left ovary. Block randomisation will be used with three varying block sizes of minimum size 4. The randomisation schedule will be produced by our statistician using the external Stata command *ralloc.*

When a participant is recruited to the trial, consecutive randomisation envelopes will be opened and the principal surgeon will be told which ovary to suspend. Only the patient's randomisation number will be recorded in the patient's operation notes. No other member of staff will be aware of women's treatment allocations.

At the end of the study, the randomisation will be unblinded for analysis and details of the ovarian suspension will be added to each patient's record.

### Incident Reporting & unblinding procedure

Adverse events will be recorded from the time of ovarian suspension until three months after surgery when the suspension results can be determined. The principal investigator will be responsible for the reporting of all serious adverse events (SAE) or suspected unexpected serious adverse reactions (SUSAR) immediately as the trial personnel become aware of an event to the chief investigator. The chief investigator should report all fatal or life-threatening events as soon as possible to the joint biomedical research unit (JBRU). This needs to be done not later than seven days after the chief investigator was first aware of the reaction. All events which are not fatal or life-threatening are also reported as soon as possible and not later than 15 days after the chief investigator was first aware of the reaction. The research and ethics committee (REA) also requires a report of all SAEs and SUSARs. The principal investigator will also follow all SAEs and SUSARs through to outcome.

In cases where patients experience SUSAR, both suspension sutures will be cut and the randomisation process will be unblinded by contacting the principal investigator or the ward sister who has a sealed randomisation envelope.

### Ethical considerations

Approval for this study was obtained from the Medical Ethical Committees of the University College Hospital, London, UK. In each patient fulfilling the inclusion criteria, written informed consent is obtained before randomisation. Women refusing participation are registered.

#### Follow-up

**Figure 1:**
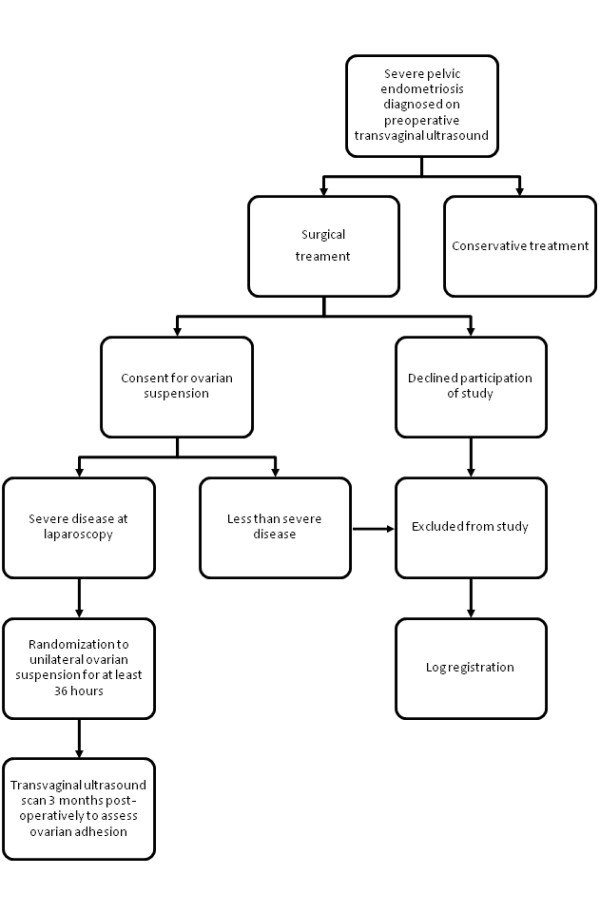
**Flowchart of ovarian suspension study**.

Three months after ovarian suspension, all patients in the study will be invited for a transvaginal ultrasound scan to assess ovarian mobility (Figure 1). Ovarian adhesions will be diagnosed in women with evidence of restricted ovarian mobility on targeted palpation using transvaginal ultrasound probe [14]. The ultrasound operators will be blinded to the details of the operative procedure and women's randomisation allocation.

#### Outcome measures

##### Primary outcome measure

The primary outcome is the prevalence of ovarian adhesion on ultrasound after surgery. The presence of ovarian adhesions will be assessed by a combination of gentle pressure with the vaginal probe and abdominal pressure with the examiners free hand as in a bimanual examination. The presence of ovarian adhesions will be diagnosed when the ovarian mobility is restricted and the ovary cannot be separated from the peritoneum of the lateral pelvic wall and/or pouch of Douglas.

##### Secondary outcome measures

Secondary outcomes are the presence, intensity and site of post-operative pain.

### Statistical analysis

Women with bilateral endometriosis will receive the normal surgical treatment with the difference that one ovary will be randomised to ovarian suspension and the other to non-suspension. The primary outcome is the binary variable of the presence of ovarian adhesions three months after laparoscopic surgery. The data will be paired binary data.

### Pilot study

A pilot study was conducted to determine the prevalence of ovarian adhesion on transvaginal ultrasound three months after routine laparoscopic treatment of severe pelvic endometriosis (without ovarian suspension).

Between 1st of March 2009 and 1st of September 2009, 16 women post laparoscopic treatment for severe endometriosis were recruited. The mean age was 34.6 years (range 22 to 51). Histology confirmed the diagnosis of endometriosis in all 16 women. Post-operatively, 11/16  (68.8% 95% CI 46.0 to 91.5) women had evidence of ovarian adhesions on TVS. 4/16 (25.0%, 95% CI 3.8 to 46.2) women had unilateral adhesions, while 7/16 (43.8%, 95% CI 19.4 to 68.1) women had adhesions involving both ovaries. The ovarian adhesion rate for each ovary was 18/32 (56.3%, 95% CI 39.1 to 73.4)

### Sample size

The prevalence of ovarian adhesion for each ovary from our pilot study was approximately 60% and this figure was used to calculate the sample size. Clinically significant improvement with ovarian suspension would be defined as a 50% reduction in the prevalence of post-operative ovarian adhesion.

The software provided by Machin et al. was used to calculate the sample size [15]. Assuming two-sided 5% significance and 80% power, 45 women would be required for the study. This calculation assumes that the response to suspension is independent to the response to non-suspension. Allowing for a possible 10% dropout during the follow up period, we plan to recruit 50 patients for the study.

## Discussion

Ovarian adhesions after surgery for pelvic endometriosis are common problems with its associated morbidity. Numerous adhesion prevention strategies, mostly based on microsurgical principles, have been reported. These include minimalising tissue trauma, meticulous hemostasis, irrigation and the use of low reactive sutures. Suspending the ovaries post-operatively has been suggested as an easy and possibly effective way of reducing ovarian adhesions. To the best of our knowledge, a larger prospective study has not been done to evaluate this. Our randomised controlled trial will provide evidence as to whether this simple surgical procedure should be included into the routine surgical treatment of women with severe pelvic endometriosis.

There is a potential risk of injury to the bowels and blood vessels during insertion of the suspension sutures. However, the chance of this occurring is likely to be very small. There has also been no reported complication as a result of ovarian suspension in previous studies.

The original study protocol was to perform a diagnostic laparoscopy to assess for ovarian adhesions three months after surgical treatment for endometriosis. However, due to problems with patient recruitment, the study protocol was modified to the current study.

Traditionally, post-operative adhesions can only be assessed by laparoscopy, but recent studies have shown that targeted palpation with ultrasound probe could be used as an alternative method to diagnose adhesion. Guerriero et al. found that the fixation of the ovary to the uterus evaluated by transvaginal ultrasonography, had a good specificity (86%) and high positive predictive value (PPV) of 81% [16]. More recently, Okaro et al. found good correlation between ovarian mobility on transvaginal ultrasound and at laparoscopy (kappa 0.81) [17]. These positive results were also shared by Holland et al [14]. Transvaginal ultrasound has proven to be an indispensible, non-invasive and inexpensive way of assessing ovarian adhesion.

In our study, a decision was made to perform the post-operative ovarian suspension for 36 to 48 hours. This was different from the duration of suspension of 4 days by Ouahba et al [13]. Our decision was based on an animal study by Harris et al. looking at the kinetics of adhesion formation of injured peritoneal surfaces [18]. They showed that a reduction of adhesion formation is achieved when separation of injured peritoneal surfaces occurs for at least 36 hours. This was in accordance with the duration of hospital stay after laparoscopic surgery for severe endometriosis. Therefore all suspension stitches can be removed before surgical discharge.

## Competing interests

ES received honoraria from Ethicon for provision of training to healthcare professionals and consultancy fees from Bayer. AC is on the advisory board for surgical innovations for which he receives an annual honorarium. AC also received support for courses and education from Storz and Johnson and Johnson and support for clinical nursing from Covidien and Lotus. The other authors declared no competing interests.

## Authors' contributions

All authors were responsible for the development of the study protocol. WH drafted the paper and has the responsibility for the logistical aspects of the trial. DJ provides supervision and writing of the draft paper. AC, ES, GP are responsible for the surgical treatment of endometriosis and executing the randomisation instructions in theatres. All authors have read and approved the final draft of this paper.

## Pre-publication history

The pre-publication history for this paper can be accessed here:

http://www.biomedcentral.com/1472-6874/11/14/prepub

## References

[B1] Cook AS Rock JA The role of laparoscopy in the treatment of endometriosis Fertil Steril 1991 55 663 680 1826275 10.1016/s0015-0282(16)54228-9

[B2] American Society for Reproductive Medicine Revised American Society for Reproductive Medicine classification of endometriosis Fertil Steril 1997 67 817 821 10.1016/S0015-0282(97)81391-X 9130884

[B3] Royal College of Obstetricians and Gynaecologists Green Top Guideline No. 24 http://www.rcog.org.uk/womens-health/clinical-guidance/investigation-and-management-endometriosis-green-top-24

[B4] Ahmad G Duffy JM Farquhar C Vail A Vandekerckhove P Watson A Wiseman D Barrier agents for adhesion prevention after gynaecological surgery Cochrane Database Syst Rev 2008 16 CD000475 10.1002/14651858.CD000475.pub2 18425865

[B5] diZerega GS Contemporary adhesion prevention Fertil Steril 1994 61 219 235 8299773 10.1016/s0015-0282(16)56507-8

[B6] Canis M Mage G Wattiez A Chapron C Pouly JL Bassil S Second-look laparoscopic cystectomy of large ovarian endometrioma Fertil Steril 1992 58 617 619 1306052 10.1016/s0015-0282(16)55274-1

[B7] Redwine DB Conservative laparoscopic excision of endometriosis by sharp dissection: life table analysis of reoperation and persistent or recurrent disease Fertil Steril 1991 56 628 634 1833246 10.1016/s0015-0282(16)54591-9

[B8] Operative Laparoscopy Study Group Postoperative adhesion development after operative laparoscopy: evaluation at early second-look procedure Fertil Steril 1991 55 700 704 1826277

[B9] Metawally M Watson A Lilford R Vandekerckhove P Fluid and pharmacological agents for adhesion prevention after gynaecological surgery Cochrane Database Syst Rev 2006 19 CD001298 10.1002/14651858.CD001298.pub3 16625541

[B10] Kamel RM Prevention of postoperative peritoneal adhesions Eur J Obstet Gynecol Reprod Biol 2010 150 111 118 10.1016/j.ejogrb.2010.02.003 20382467

[B11] Cutner AS Lazanakis MS Saridogan E Laparoscopic ovarian suspension to facilitate surgery for advanced endometriosis Fertil Steril 2004 82 702 704 10.1016/j.fertnstert.2004.02.115 15374717

[B12] Abuzeid MI Ashraf M Shamma FN Temporary ovarian suspension at laparoscopy for prevention of adhesions J Am Assoc Gynecol Laparosc 2002 9 98 102 10.1016/S1074-3804(05)60114-4 11821616

[B13] Ouahba J Madelenat P Poncelet C Transient abdominal ovariopexy for adhesion prevention in patients who underwent surgery for severe pelvic endometriosis Fertil Steril 2004 82 1407 1411 10.1016/j.fertnstert.2004.03.060 15533368

[B14] Holland TK Yazbek J Cutner A Saridogan E Hoo WL Jurkovic D Value of transvaginal ultrasound in assessing severity of pelvic endometriosis Ultrasound Obstet Gynecol 2010 36 241 248 20503231 10.1002/uog.7689

[B15] Machin D Campbell MJ Tan SB Tan SH Sample Size Tables for Clinical Studies. Comparing paired groups of binary, ordered categorical and continuous outcomes 2009 Third Wiley-Blackwell 69 83

[B16] Guerriero S Ajossa S Lai MP Mais V Paoletti AM Melis GB Transvaginal ultrasonography in the diagnosis of pelvic adhesions Hum Reprod 1997 12 2649 2653 10.1093/humrep/12.12.2649 9455829

[B17] Okaro E Condous G Khalid A Timmerman D Ameye L Huffel SV Bourne T The use of ultrasound-based 'soft markers' for the prediction of pelvic pathology in women with chronic pelvic pain--can we reduce the need for laparoscopy? BJOG 2006 113 251 256 10.1111/j.1471-0528.2006.00849.x 16487194

[B18] Harris ES Morgan RF Rodeheaver GT Analysis of the kinetics of peritoneal adhesion formation in the rat and evaluation of potential antiadhesive agents Surgery 1995 117 663 669 10.1016/S0039-6060(95)80010-7 7539943

